# ICan, an Internet-based intervention to reduce cannabis use: study protocol for a randomized controlled trial

**DOI:** 10.1186/s13063-020-04962-3

**Published:** 2021-01-06

**Authors:** Marleen I. A. Olthof, Matthijs Blankers, Margriet W. van Laar, Anna E. Goudriaan

**Affiliations:** 1 Trimbos Institute, Netherlands Institute of Mental Health and Addiction, Utrecht, The Netherlands; 2 Amsterdam UMC, Department of Psychiatry, University of Amsterdam, Amsterdam, The Netherlands; 3 Arkin Mental Health Care, Amsterdam, The Netherlands; 4 Amsterdam Public Health Research Institute, Amsterdam, The Netherlands

**Keywords:** Cannabis, eHealth, SBIRT, Web-based program, Intervention, Substance use disorder

## Abstract

**Background:**

Heavy cannabis use is associated with adverse physical and mental health effects. Despite available effective treatments, the majority of heavy cannabis users does not seek professional help. Web-based interventions can provide an alternative for cannabis users who are reluctant to seek professional help. Several web-based cannabis interventions are effective in reducing cannabis use; however, the effect sizes are typically small and attrition rates are typically high. This suggests that web-based programs can be an effective cannabis use intervention for some, while others may need additional substance use treatment after completing a web-based intervention. Therefore, it is important that web-based interventions do not solely focus on reducing cannabis use, but also on improving attitudes towards substance use treatment. The Screening Brief Intervention and Referral to Treatment (SBIRT) approach appears to be well suited for the purpose of reducing cannabis use and improving substance use treatment utilization. Based on the SBIRT approach—and based on cognitive behavioral therapy (CBT) and motivational interviewing (MI)—we developed the Internet-based cannabis reduction intervention ICan.

**Methods/design:**

This protocol paper presents the design of a randomized controlled trial (RCT) in which we evaluate the effectiveness of the ICan intervention compared to four online modules of educational information on cannabis in a sample of Dutch frequent cannabis users. The primary outcome measure is frequency of cannabis use. Secondary outcome measures include the quantity of cannabis used (grams), the attitudes towards seeking help and the number of participants who enter specialized treatment services for cannabis use-related problems.

**Discussion:**

To the best of our knowledge, ICan is the first Internet-based intervention for cannabis users that combines screening, a brief intervention—based on CBT and MI—and referral to treatment options.

**Trial registration:**

The study is registered in the Netherlands Trial Register; identifier NL7668. Registered on 17 April 2019.

## Background

Heavy cannabis use in adolescence and young adulthood is associated with various adverse physical and mental health effects [1]. These effects include cognitive impairment and an increased risk of depressive symptoms and suicidal ideation [1]. Heavy cannabis users are at risk for dependence [2, 3]. A longitudinal study of a cohort of (near) daily cannabis users found that almost 40% of the (near) daily cannabis users developed cannabis dependence (DSM-IV) [3].

Treatment programs based on cognitive behavioral therapy (CBT), motivational interviewing (MI), and contingency management are effective in reducing cannabis use [4]. However, the majority of frequent cannabis users does not seek professional help [5, 6]. In the Netherlands, the number of people receiving treatment for cannabis use-related problems increased from 2001 to 2010 and then stabilized until 2015 [7]. In 2015, 11,000 people received treatment for cannabis use-related problems, while according to the most recent estimates (2007–2009) 30,000 people met the criteria for cannabis dependence [7, 8]. Several studies have identified possible explanations for the low numbers of cannabis users entering treatment specifically, and for substance users in general. Commonly reported barriers for seeking treatment are the desire to solve one’s own problems, the feeling that treatment is not necessary, not being ready to stop using cannabis, being unaware of treatments options, not being able to attend treatment during office hours, and stigma associated with substance use disorder treatment [9, 10].

Internet-based programs can overcome some of these barriers and thereby provide an alternative for frequent cannabis users who are unwilling to enter substance use treatment [11]. Internet-based programs are characterized by a high degree of anonymity; this can minimize the fear of being stigmatized [12]. Besides, they are easily accessible, as users can access the programs from any location at any time of day. In addition, the programs can be followed at their own pace, which heightens (perceived) feasibility of following the program. Internet-based programs require less therapist time per patient than face-to-face treatments; therefore, they may also be more cost-effective [13].

Studies show that Internet-based programs for cannabis users are effective. Boumparis et al. recently published a systematic review with meta-analyses on digital prevention and treatment interventions to reduce cannabis use [14]. The meta-analyses showed a small but significant effect in favor of digital interventions compared to control conditions (waiting list, psycho-education or assessment only) [14]. These results are in line with results found in earlier meta-analyses on Internet and computer-based interventions for cannabis use [15–17].

Thus, digital interventions for cannabis users can be effective and have the potential to overcome some commonly reported barriers to treatment-seeking, although effect sizes are generally small. Therefore, they can possibly play an important role in bridging the cannabis use disorder treatment gap. To our knowledge, four Internet-based cannabis reduction programs for the non-clinical population of frequent cannabis users have been evaluated in randomized controlled trials. The first program, a German program called *Quit the shit* is based on the principles of self-regulation and self-control [18]. The 50-day program has a solution-focused approach and includes weekly interaction with a therapist through instant messaging.

The second program, the Australian program *Reduce your Use* consists of 6 modules based on cognitive, behavioral, and motivational principles [19]. The program is fully self-guided; the participants can go through the modules at their own pace.

The third program, the Swiss program *Can Reduce*, is based on CBT, MI, and behavioral self-management [20]. The effectiveness of the program with and without guidance has been tested. The guidance consists of two chat sessions with a trained counselor. The chat sessions have a duration of 20–30 min.

The fourth program, the Swedish program *Cannabishjälpen*, is also based on CBT and MI principles [21]. The program consists of 13 modules. Participants are advised to complete one or two modules per week. At the beginning of the program, a therapist sends a welcome message to the participant including personalized feedback on the baseline assessment. Throughout the program, the participant can contact the therapist if desired.

The Cannabishjälpen program and the unguided version of the Can Reduce program were not effective in reducing cannabis use frequency (compared to the waiting list control condition) [20, 21]. The other programs—*Quit the Shit*, *Reduce your Use* and the guided version of *Can Reduce*—were effective in reducing cannabis use [18–20]. However, the effect sizes were small and attrition was high. These small effect sizes and high attrition rates suggest that online programs can provide an alternative for some, but not for all cannabis users who are reluctant to enter substance use treatment. Therefore, it seems important that online programs do not solely focus on reducing cannabis use, but also on improving attitudes towards substance use treatment. If a cannabis user fails to reduce his use after completing the online program, he may be willing to start/engage in substance use disorder treatment.

The Screening Brief Intervention and Referral to Treatment (SBIRT) approach appears to be well suited for the purpose of improving substance use treatment utilization. The SBIRT approach was developed in the 1960s [22]. The SBIRT approach enables universal screening in a variety of settings, targeting not only those who are already dependent but also those who are not seeking help for their substance use [23]. The screening procedure typically results in three possible outcomes: no risk, moderate risk, or high risk for substance use problems. Substance users at moderate risk for substance use problems receive a brief intervention. The brief intervention usually consists of one or more sessions with a health care professional. The goal of these sessions is to raise awareness about the risks associated with the substance use and to increase motivation to reduce or stop this behavior [23]. Substance users at high risk for cannabis use problems are referred to specialized substance use treatment. The main goal of the referral to treatment is to identify an appropriate treatment program and to facilitate participation of the substance user in the program [23]. The SBIRT approach seems suitable to be computerized.

Based on the SBIRT approach and based on cognitive behavioral therapy and motivational interviewing, we developed the Internet-based cannabis reduction intervention ICan. ICan is an easy to use progressive web app. ICan includes adherence focused guidance to minimize drop-out rates. Users of the ICan app receive weekly WhatsApp messages from a coach to encourage them to use the app. The guidance is minimal to ensure that the intervention remains easily accessible. This protocol paper presents the design of the randomized controlled trial (RCT) in which we evaluate the effectiveness of the ICan intervention.

## Methods

### Aims and hypotheses

The aim of this study is to test the effectiveness of the Internet-based intervention ICan compared to four online modules of educational information on cannabis in a sample of Dutch frequent cannabis users. We address the following research questions: (1) Is the ICan intervention more effective in reducing cannabis use than the control condition? (2) Is the ICan intervention more effective in improving positive attitudes towards seeking professional help for cannabis use-related problems than the control condition?

### Study design

A single blind randomized controlled trial will be carried out with a duration of 6 months in an online setting. The trial will be two armed (ICan intervention x four online modules of educational information on cannabis). Participants will be assessed on cannabis-related outcome measures at T0 (baseline, before randomization), T1 (6 weeks post randomization), T2 (3 months post randomization), and T3 (6 months post randomization). The trial will be conducted and reported according to the most recent version of the Consolidated Standards of Reporting Trials (CONSORT) guidelines [24]. The study is registered in the Netherlands Trial Register; identifier NL7668. Ethical approval to carry out this study was obtained from an accredited medical research and ethics committee in the Netherlands (Medical Research Ethics Committees United, NL67449.100.18). The study is designed and will be performed in compliance with the Declaration of Helsinki, seventh revision.

### Study procedures

**Fig. 1:**
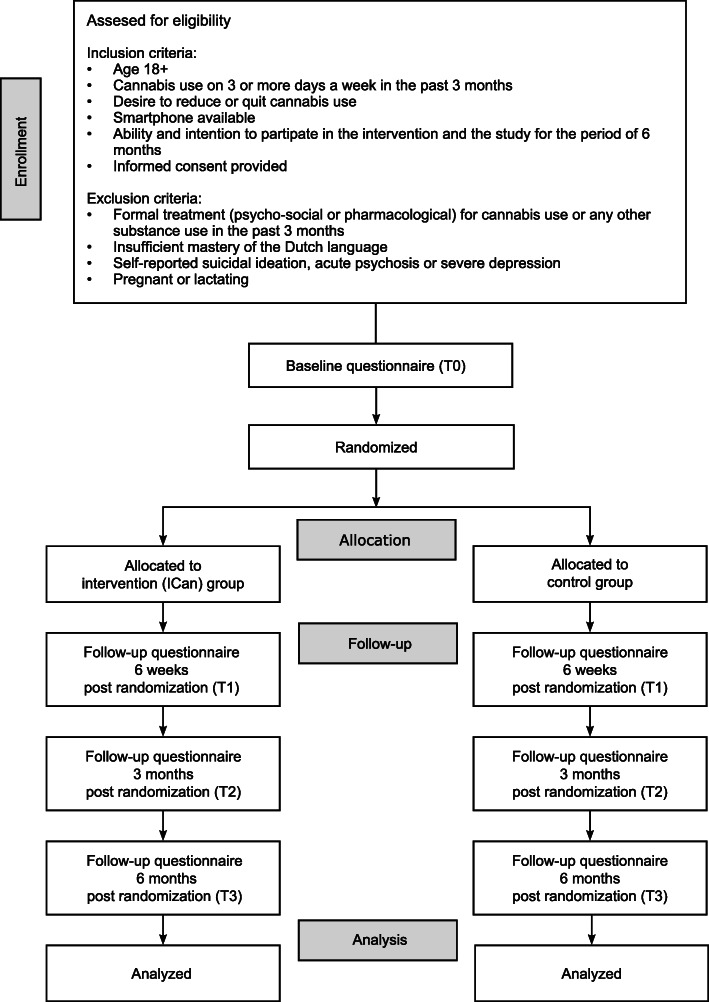
Trial flow

Figure 1 shows the CONSORT flow diagram of the trial. Applicants interested to participate fill out an online screening questionnaire to determine if they meet all of the inclusion criteria and none of the exclusion criteria. Applicants who are eligible to participate receive the patient information letter and the informed consent form. Participants have up to 30 days to decide if they want to participate. If they have any questions regarding the study or intervention, they can contact a member of the research team by phone, email, or face-to-face. They can also contact an independent expert whose contact details are listed in the patient information letter. Applicants who decide to participate in the study are asked for necessary personal data. After the participants have sent us their signed informed consent form digitally, they are directed to the baseline questionnaire. The electronic data capture platform Castor will be used for the randomization and allocation procedure and to conduct the online questionnaires. The research data are stored separately from the participants’ personal data. Only the four authors of this study protocol will have access to the keys to join the research data tables with the personal data tables. After the participants have completed the baseline questionnaire, they will be allocated to one of two trial arms (1:1) using variable block randomization. Participants will be informed that they will be assigned to one of two programs, both focusing on cannabis moderation. Depending on the outcome of the allocation procedure, an email will be sent to the participants containing an access code to either the ICan intervention or the online control program (four online modules of educational information on cannabis). Participants will be blind to the condition they are in.

The follow-up measurements will take place 6 weeks, 3 months, and 6 months post randomization. Self-reported outcome measures are used to reduce the risk of experimenter bias. If participants do not complete the online follow-up questionnaires, they will first receive an automatic email reminder; subsequently, they will receive WhatsApp (audio) messages to encourage them to fill in the questionnaires. All participants receive the same (audio) messages to reduce the risk of bias.

After completing the 3 months’ follow-up questionnaire and after completing the 6 months’ follow-up questionnaire, the participants will receive a €20 gift card by email. Even if participants discontinue their use of the intervention prematurely, they will be followed up. Only if participants explicitly state that they do not want to participate in the study anymore, data collection will be stopped. After completing the last follow-up questionnaire, the participants are informed about the condition they were allocated to. If desired, they can cross over to the other condition.

All spontaneously reported adverse events will be recorded. All serious adverse events will be reported to the accredited MREC (Medical Research Ethics Committee) that approved the protocol. Given the limited risks associated with a text-based self-help intervention, no Data Safety Monitoring Board or Safety Committee will be established for this study.

### Participants

#### Recruitment

The population base from which the subjects will be drawn are Dutch non-treatment seeking frequent cannabis users, meeting all inclusion and none of the exclusion criteria. For the purpose of the trial, a website is created containing information about the study and the possibility to register as a potential participant. To ensure the recruitment of the planned number of participants targeted Facebook and Instagram campaigns will be used. We have experienced that this is a very effective strategy for the recruitment of cannabis users. In 2018, we conducted an exploratory online survey among frequent cannabis users [25]. In just a few months the online campaigns resulted in the recruitment of more than 1000 eligible participants for the survey study. In this study protocol, “he” is used to refer to a participant; however, where “he” is stated, “he or she” is meant.

#### In- and exclusion criteria

In order to be eligible to participate in the study, a subject must meet all of the following criteria:

- Age 18+
- Cannabis use on 3 or more days a week in the past 3 months
- Desire to reduce or quit cannabis use
- Smartphone available
- Ability and intention to participate in the training and study for the period of 6 months
- Informed consent provided

A potential subject who meets any of the following criteria will be excluded form participation in this study:

- Formal treatment (psycho-social or pharmacological) for cannabis use or any other substance use in the past 3 months
- Insufficient mastery of the Dutch language
- Self-reported suicidal ideation, acute psychosis, or severe depression
- Pregnant or lactating

#### Sample size

For the RCT, conventional power (1-*β* = 0.80) and levels of statistical significance are chosen (*α* = .05, 2-sided). The meta-analysis conducted by Boumparis et al. revealed a small but significant effect in favor of digital prevention interventions compared to control conditions, an effect size of g = 0.33 was found [14]. However, the effect size of our online SBIRT program is expected to be somewhat larger due to the fact that the brief intervention is only one component of the program. The referral to treatment component may also have some effect on cannabis consumption. The referral to treatment component consists of information about specialized treatment services for substance use problems. Participants are encouraged to utilize these services. When participants enter treatment, it is likely that this will also have an effect on their cannabis consumption. Therefore, we expect an effect size of *d* = 0.4 or more. G*Power was used to calculate the required number of participants. The power calculation is based on 2-sided-tests, *d* = 0.40 corresponds with a sample size of at least 2 × 100 participants. Assuming a 25% non-response at 6 months’ follow-up, we aim to include at least (100/(100–25)) × 100 × 2 = 267 participants. In case the dropout is higher than expected, this number may be adjusted upwards.

### Intervention condition

The experimental ICan intervention is designed according to the SAMHSA Screening Brief Intervention and Referral to Treatment (SBIRT) framework and is based on cognitive behavioral therapy (CBT) and motivational interviewing (MI). The participant receives an e-mail with an invitation to create an account. The ICan intervention includes adherence-focused guidance. From the moment the participant creates an account, he receives a weekly WhatsApp message from a coach to encourage him to use the app. In the first message(s), the participant is encouraged to complete the self-test and to create the reduce/quit plan. In the subsequent message(s), the participant is encouraged to fill in the consumption diary regularly. Besides the WhatsApp messages, the participant receives motivational push notifications. The participant can set up reminders to fill in the consumption diary. He can indicate how often he would like to receive the reminders and at what time. After 4 weeks, the participant is asked if he is satisfied with the progress he has made so far. If he is not satisfied he is referred to specialized treatment services (see part 4). After these 4 weeks, the WhatsApp messages are discontinued—unless the participant explicitly indicates that he wants to keep receiving the messages. In that case, he receives a weekly message in which he is praised for his achievements in the app—for example completing the additional problem solving exercise, posting a message on the peer support platform, or filling in the consumption diary. The participant keeps receiving these messages as long as he is actively using the app.

#### ICan part 1: screening

The screening procedure consists of four short self-tests.

- *How is your cannabis consumption compared to others?* The participant answers a set of questions about his cannabis use. Based on his answers, personalized normative feedback (PNF) is given. PNF aims to correct the misperception that the use of substances is common among people of the same peer group as the participant. The amount of money that the participant can save by quitting his cannabis use is calculated (per week, month, and year).
- *What type of cannabis user are you?* The second test is based on the Marijuana Motives Measure [26]. The test assesses six motives for using cannabis: enhancement, coping, social, conformity, expansion, and routine. The average score on the six subscales/motives is provided to the participant.
- *How risky is your cannabis use?* This third test is based on the ASSIST [27]. The participant answers six questions about his cannabis use in the past 3 months. Based on his answers, a personalized feedback report about the risks associated with his cannabis use is provided. Participants at risk for cannabis use-related problems are advised to reduce or quit their cannabis use. The emphasis lies on the health gains that can be achieved by reducing or quitting their cannabis use. Participants at severe risk for cannabis use-related problems are advised to seek specialized treatment. However, if a participant is still hesitant about reducing his cannabis use and/or seeking help, he also gets the option to complete the online training (first).
- *Are you “addicted” to cannabis?* The fourth and last test is based on the Cannabis Use Disorders Identification Test Revised (CUDIT-R) [28]. The participant answers 8 questions about his cannabis use in the past 6 months. Based on his answers, feedback is given about the risk for cannabis use disorder. Cannabis users at moderate or severe risk for cannabis use disorder are advised to reduce or stop their cannabis use. They are referred to the ICan (web) app for further information about cannabis use disorder and specialized treatment services.

#### ICan part 2: brief intervention (I)

After the self-test is completed, an introduction slider is displayed with the main features of the app. The main features are the homepage, consumption diary, reduce/quit plan, personal profile page, peer support platform, and treatment info pages.

The brief intervention component helps participants to create a personalized plan to stop or reduce their cannabis use step by step. The steps (with the exception of step 3b) are based on the protocol for brief cognitive behavioral treatment for substance use disorders [29].

- Step 1: Motivation to change. The participant first lists the advantages of using cannabis; then, he lists the disadvantages of using cannabis and the advantages of reducing or quitting. Based on these lists, the participant decides whether he wants to continue his cannabis use or wants to reduce/quit.
- Step 2: Setting a goal. The participant determines whether he wants to reduce or quit his use and picks a quit date.
- Step 3a: How to achieve your goal. The participant identifies high-risk situations: situations, thoughts, and/or feelings that trigger a strong craving to use cannabis. A short animation video explains how self-control strategies can be applied to prevent cannabis use. Subsequently, the participant describes how he will apply these strategies to deal with high-risk situations in the future.
- Step 3b: How to solve problems. If a participant experiences problems (e.g., financial problems) that may negatively impact his ability to reduce or stop cannabis use, he can do an additional exercise in which he learns how to solve problems step-by-step.
- Step 4: Social support. The participant describes how his friends, acquaintances, and/or loved ones can support him during his reduce/quit attempt. He lists the names of the persons he wants to ask for support.
- Step 5: Withdrawal symptoms, craving, and relapse. The participant is informed about withdrawal symptoms. A short animation video provides tips on how to deal with craving. The participant describes how he wants to deal with craving and relapses.
- Step 6: Your personal (quit) plan. By completing step 1 through 5, the participant has created a personal reduce/quit plan, this plan is summarized in step 6.

#### ICan part 3: brief intervention (II)

From the set (quit) date, the participant is encouraged to stick to his (quit) plan. Based on the entries in the cannabis consumption diary, automatic tailored feedback is given. On the personal profile page, a bar graph shows the progress made by the participant. Icons are used to show in which situations (time of day, type of activity, in the presence of which emotion/feeling, alone or with others) the participant is most likely to experience craving and in which situations he is most likely to use cannabis. The number of days without cannabis use and the amount of money saved are also displayed on this page. The participant is advised to stick to his personal (quit) plan for at least 4 weeks.

After 4 weeks, the participant evaluates if he is satisfied with the progress he has made so far. If desired, a new goal can be set. Participants who fail to stick to their plan are referred to specialized treatment services (see part 4). If a participant is reluctant to engage in specialized treatment, he can continue to use the ICan (web)app.

#### ICan part 4: referral to treatment

The referral to treatment component consists of comprehensive information on specialized treatment services for substance use problems. The information is summarized in a short animation video. Participants can easily check which treatments are offered nearby based on their location/zip code.

#### Peer support platform

On the peer support platform, participants can write messages to motivate and support each other.

#### Badges

The participants can earn badges for their achievements in the app. The earned badges are displayed on the badge page.

### Control condition

The control condition consists of four online modules of educational information on cannabis. In the first module, some background information about cannabis is provided. This includes information on the physical and mental effects of cannabis, commonly used consumption methods and cannabis and the law. In the second module, the risks associated with cannabis use are listed. Some tips on how to reduce these risks are given. In the third module, general information about cannabis use disorder is provided. The difference between physical and psychological dependence is explained. The negative social, financial, and health consequences of cannabis use disorder are described, and the DSM-5 criteria for cannabis use disorder are presented. In the last module, some general tips on how to reduce or quit cannabis use are provided. The information used in the four online modules is based on the websites www.drugsinfo.nl and www.drugsenuitgaan.nl. The general tips on how to reduce or quit cannabis use are from an existing information brochure about cannabis use and psychosis [30]. Since the control condition consists of information that is currently available online for non-treatment seeking frequent cannabis users, we considered this to be the standard care (or treatment-as-usual).

### Outcome measures

#### Primary measures

**Fig. 2:**
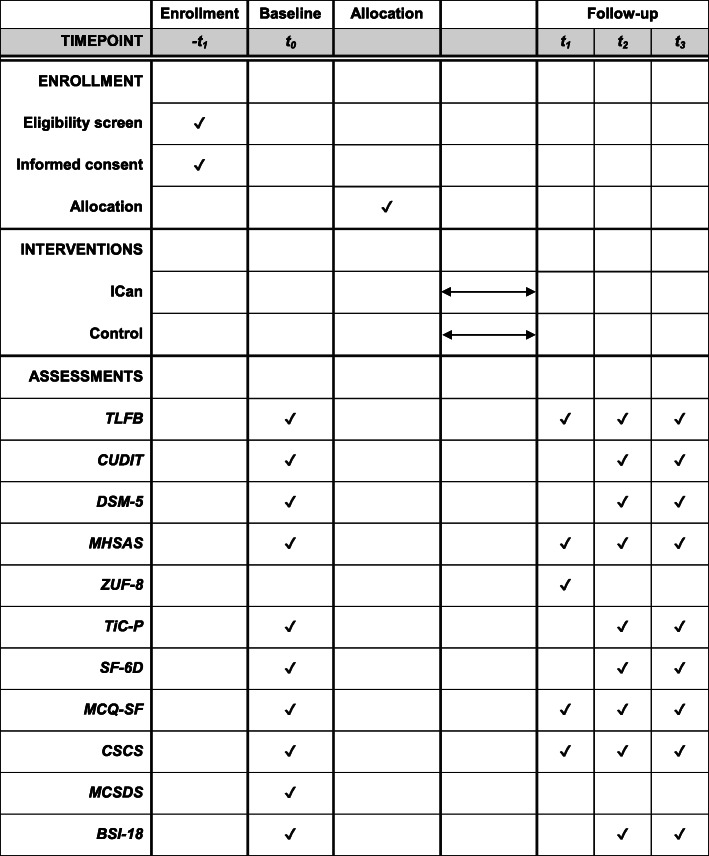
SPIRIT diagram. Schedule of enrollment, allocation, interventions, and assessments. TLFB, Timeline Follow-back; CUDIT, Cannabis Use Disorders Identification Test; DSM-5, number of DSM-5 criteria met (self-reported); MHSAS, Mental Help Seeking Attitudes Scale; ZUF-8, Fragebogen zur Messung der Patientenzufriedenheit; TiC-P, Trimbos and iMTA questionnaire on Costs associated with Pychiatric illness; SF-6D, ShortForm 6 Dimensions; MCQ-SF, Marijuana Craving Questionnaire Short Form; CSCS, Cannabis Self-Concept Scale; MCSDS, Marlowe-Crowne Social Desirability Scale; BSI-18, Brief Symptom Inventory 18

Figure 2 shows the SPIRIT diagram of the trial. The primary outcome measure is the number of cannabis use days in the past 7 days, assessed 6 months post randomization using the Timeline Follow-back (TLFB) method [31]. The TLFB method is originally developed as an interview to assess self-reported alcohol consumption [31]. Growing evidence suggests that the TLFB is a psychometrically sound measure for the assessment of licit and illicit substance use [32]. Self-reports on cannabis use measured using the TLFB method show high test-retest reliability [33]. TLFB reports administered online are consistent with TLFB reports administered face-to-face [34].

#### Secondary measures

##### Cannabis use, cannabis use disorder, and cannabis use-related problems

The following secondary outcome measures will be applied (see also Fig. 2):

- The number of cannabis use days in the past 7 days assessed using the TLFB method 6 weeks post randomization and 3 months post randomization.
- The quantity of cannabis used (grams) in the past 7 days assessed using the TLFB method 6 weeks post randomization, 3 months post randomization, and 6 months post randomization. Participants are first asked to indicate how many joints they usually make from a single gram of cannabis. Next, they are asked to fill out the calendar. They indicate, for each of the 7 days listed on the calendar, how many joints they smoked. If they smoked only half a joint on a particular day, they are asked to fill-in ½. Based on the number of joints made from a single gram and the number of joints smoked, the quantity of cannabis used (grams) is calculated. Van der Pol et al. [35] validated different self-report measures on cannabis dose against objective measures. Self-reported number of joints per gram was one of the least problematic options.
- Cannabis use-related problems as measured using the Cannabis Use Disorders Identification Test (CUDIT), a 10-item questionnaire based on the Alcohol Use Disorders Identification Test [36].
- The cannabis use disorder severity, as measured by the number of self-reported DSM-5 criteria met [37].
- Number of self-reported previous serious attempts to reduce or quit cannabis use. A serious quit attempt is defined as an attempt that lasted at least 24 h.

##### Treatment seeking behavior and attitudes

f) Help seeking attitudes as measured by the Mental Help Seeking Attitudes Scale (MHSAS) [38]. MHSAS is a 9-item questionnaire designed to assess the overall attitude towards seeking help (unfavorable–favorable) from a mental health professional if one had to deal with a mental health problem. Each item is rated on a 7-point-scale. We adapted the questionnaire to measure attitudes towards seeking professional help specifically for cannabis use-related problems.
g) Based on Ajzen’s Theory of Planned behavior, we constructed a questionnaire to assess intention, social norm, perceived behavioral control, and attitudes towards seeking professional help for cannabis use-related problems.
h) The Trimbos and iMTA questionnaire on Costs associated with Pychiatric illness (TiC-P) will be used to measure the number of participants who entered specialized treatment services for cannabis use-related problems 3 months and 6 months post randomization [39]. Some questions are added to determine whether participants visited a specific health care professional for cannabis use-related problems, for other problems or for both. Complementary, participants are presented with a list of more informal types of help (e.g., seeking help from a religious confidant, seeking help from a loved one, seeking help online) and are asked to indicate whether they used any of these types of help in the past 3 months.

##### Intervention satisfaction

i) Intervention satisfaction is measured with the Dutch translation of the Fragebogen zur Messung der Patientenzufriedenheit (ZUF-8) [40]. The ZUF-8 is a free-to-use German version of the Client Satisfaction Questionnaire (CSQ-8). The questionnaire consists of 8 items; each item is rated on a 4-point-scale. The ZUF-8 has good psychometric properties [41].

##### Cost-effectiveness

j) The Trimbos and iMTA questionnaire on Costs associated with Pychiatric illness will be used to measure health care utilization and production loss (TiC-P) [39].
k) Quality of life will be measured using the SF-6D [42].

##### Mediators and other measures

The following instruments will be applied to assess their potential role as intervention effect mediator:

l) The number of self-reported cannabis withdrawal symptoms.
m) Craving for cannabis will be assessed using the Marijuana Craving Questionnaire Short Form (MCQ-SF) [43].
n) The Cannabis Self-Concept Scale (CSCS) will be included to assess identification with cannabis as part of one’s personality or identity [44].
o) Self-efficacy to become or stay a non-cannabis smoker will be measured by 6 items used in previous research [45].
p) The Marlowe-Crowne Social Desirability Scale (MCSDS) will be included to measure if the respondent has the tendency to give socially desirable responses [46].
q) The Brief Symptom Inventory 18 (BSI-18) will be included to assess symptoms of anxiety and depression [47].
r) Self-reported substance use (tobacco, alcohol, cocaine, amphetamine, inhalants, sedatives, hallucinogens, opioids, and other drugs).
s) Utilization measures will be collected during the use of the intervention: number of logins, number of page views, time spent logged in, and use of major content elements.
t) Demographic characteristics of the participant will be collected such as age, sex, and level of education.
u) Knowledge obtained about CBT principles will be measured with a self-constructed 5-item questionnaire.
v) Knowledge obtained about treatment options/referral to treatment will be measured with a self-constructed 5-item questionnaire.
w) The effect of the coronavirus and the coronavirus measures on cannabis use and attempts to reduce/quit cannabis use will be measured with a self-constructed 7-item questionnaire.

### Statistical analyses

Conventions with regard to the analysis of the data and reporting of the study will be followed, in this case the CONSORT statement guidelines [24]. We recognize the importance of open data initiatives and intend to—under conditions—open the anonymized datasets collected in this research project to whoever is professionally interested and entitled to use these data, as long as it is acceptable to the research participants and ethical standards. Generalized linear mixed models (GLMM) will be applied to the primary and secondary outcome measures. Time will be modeled as a categorical variable. Depending on the data, we will use random slopes. When there is a lot of variation in the number of cannabis use days at baseline—we expect this will be the case—then we will at least fit a random intercept. The link function of the GLMM will be chosen depending on the data: most likely this will be a binomial distribution in the case of dichotomous outcome measures, Gaussian in the case of normally distributed data and (overdispersed) Poisson or negative binomial for count data. Covariates in the model will be variables that differ at baseline (*p* < .05) and the MCSDS. Missing data is expected to be missing at random and will be handled using the Multivariate Imputation via Chained Equations (MICE) package in R.

## Discussion

This paper describes the protocol to test the effectiveness of an Internet-based intervention with adherence-focused guidance on reducing cannabis use and improving positive attitudes towards seeking professional help in a sample of frequent cannabis users via a two-armed randomized controlled trial. To the best of our knowledge, this is the first Internet-based intervention for cannabis users that combines screening, a brief intervention based on CBT and MI, and referral to treatment.

All outcome measures in this study are based on self-report questionnaires, not on biological measures such as hair or urine samples. It is not possible to determine whether participants will truthfully report their cannabis use or will give socially desirable answers. As mentioned before, the TLFB method is a psychometrically sound measure for the assessment of licit and illicit substance use [32]. Research has shown that the TLFB method for the detection of substance use, including cannabis use, has high levels of overall agreement with biological measures [32]. To control for potential socially desirable answers, the MCSDS will be included.

A number of studies have been done on the effectiveness of SBIRT programs. Most of these studies focus on the screening and brief intervention components of the programs. A study initiated by the Substance Abuse and Mental Health Services Administration tested the effectiveness of an SBIRT program for alcohol and illicit drug use, implemented in a range of medical settings [48]. More than 450.000 patients were screened, 22.7% of them screened positive for risky alcohol use and/or illicit drug use. Depending on the severity of use and associated risk, people were recommended for a brief intervention, brief treatment, or referral to specialty treatment. At 6 months’ follow up, rates of drug use were 67% lower [48]. This study suggests that brief interventions as part of SBIRT programs can significantly reduce substance use. However, a systematic review concluded that insufficient evidence exists to determine whether brief interventions as part of the SBIRT program are effective or ineffective in reducing substance use [49]. Besides, research addressing the effectiveness of the referral to treatment component is still scarce [23, 49]. In conclusion, more research regarding the effectiveness of SBIRT programs for the reduction of substance use seems needed. The SBIRT programs included in the systematic review were mainly face-to-face interventions. However, SBIRT programs seem highly suitable to be computerized.

Studies on computerized interventions for anxiety, depression, and problematic alcohol use show that interventions guided by a coach or therapist are more effective than unguided interventions [50–53]. High attrition rates in online interventions are common, especially when there is no guidance from a coach or therapist [50]. These findings suggest that guided interventions are preferred over unguided interventions. However, unguided self-help interventions are often offered free of charge and can be used without a GP referral. This makes them easily accessible, which may be in particular important for cannabis users who are still in doubt about reducing their cannabis use. We have taken these considerations into account and decided to offer adherence focused guidance to minimize drop-out rates. The guidance is minimal to ensure that the program remains easily accessible. The present trial will improve scientific knowledge regarding the effectiveness of Internet-based SBIRT programs with adherence focused guidance for frequent cannabis users.

### Study status

Protocol version 1.1. Recruitment began on 23 December 2019 and was completed on 26 May 2020.

## Data Availability

The anonymized datasets used and/or analyzed during the current study are available from the corresponding author on reasonable request.

## Abbreviations

CBT: Cognitive behavioral therapy
MI: Motivational interviewing
SBIRT: Screening, Brief Intervention and Referral to Treatment
